# Effect of Mobile Phone App–Based Interventions on Quality of Life and Psychological Symptoms Among Adult Cancer Survivors: Systematic Review and Meta-analysis of Randomized Controlled Trials

**DOI:** 10.2196/39799

**Published:** 2022-12-19

**Authors:** Minghui Qin, Bo Chen, Shaohua Sun, Xiaodong Liu

**Affiliations:** 1 Department of Traditional Chinese Medicine, Xiangyang Central Hospital, Affiliated Hospital of Hubei University of Art and Science Xiangyang China; 2 Center for Clinical Evidence-Based and Translational Medicine, Xiangyang Central Hospital, Affiliated Hospital of Hubei University of Arts and Science Xiangyang China; 3 Department of Oncology, Suizhou Central Hospital, Hubei University of Medicine Suizhou China; 4 Information Center Xiangyang Central Hospital Affiliated Hospital of Hubei University of Art and Science Xiangyang China

**Keywords:** mobile health app, mHealth app, quality of life, psychological symptoms, cancer survivors, systematic review and meta-analysis, mobile phone

## Abstract

**Background:**

Most patients with cancer experience psychological or physical distress, which can adversely affect their quality of life (QOL). Smartphone app interventions are increasingly being used to improve QOL and psychological outcomes in patients with cancer. However, there is insufficient evidence regarding the effect of this type of intervention, with conflicting results in the literature.

**Objective:**

In this systematic review and meta-analysis, we investigated the effectiveness of mobile phone app interventions on QOL and psychological outcomes in adult patients with cancer, with a special focus on intervention duration, type of cancer, intervention theory, treatment strategy, and intervention delivery format.

**Methods:**

We conducted a literature search of PubMed, Web of Science, the Cochrane Library, Embase, Scopus, China National Knowledge Infrastructure, and WanFang to identify studies involving apps that focused on cancer survivors and QOL or psychological symptoms published from inception to October 30, 2022. We selected only randomized controlled trials that met the inclusion criteria and performed systematic review and meta-analysis. The standardized mean difference (SMD) with a 95% CI was pooled when needed. Sensitivity and subgroup analyses were also conducted.

**Results:**

In total, 30 randomized controlled trials with a total of 5353 participants were included in this meta-analysis. Compared with routine care, app interventions might improve QOL (SMD=0.39, 95% CI 0.27-0.51; *P*<.001); enhance self-efficacy (SMD=0.15, 95% CI 0.02-0.29; *P*=.03); and alleviate anxiety (SMD=−0.64, 95% CI −0.73 to −0.56; *P*<.001), depression (SMD=−0.33, 95% CI −0.58 to −0.08; *P*=.009), and distress (SMD=−0.34, 95% CI −0.61 to −0.08; *P*=.01). Short-term (duration of ≤3 months), physician-patient interaction (2-way communication using a smartphone app), and cognitive behavioral therapy interventions might be the most effective for improving QOL and alleviating adverse psychological effects.

**Conclusions:**

Our study showed that interventions using mobile health apps might improve QOL and self-efficacy as well as alleviate anxiety, depression, and distress in adult cancer survivors. However, these results should be interpreted with caution because of the heterogeneity of the interventions and the study design. More rigorous trials are warranted to confirm the suitable duration and validate the different intervention theories as well as address methodological flaws in previous studies.

**Trial Registration:**

PROSPERO CRD42022370599; https://www.crd.york.ac.uk/PROSPERO/display_record.php?RecordID=370599

## Introduction

### Background

Worldwide, the number of new cancer cases diagnosed each year is rapidly increasing, from 14.1 million in 2012 to an estimated 21.6 million in 2030 [1]. With advancements in early detection and clinical treatment techniques, these patients now have a better prognosis and longer life expectancy [2]. However, approximately 30% to 40% of patients with cancer have at least one psychological or physical symptom, such as anxiety, depression, or distress [3-5], and up to 50% of women diagnosed with breast cancer experience psychological issues at some point in their illness [6], which may negatively affect their quality of life (QOL) and make them more stressed [7,8].

Although psychological problems are common in patients with cancer, they are not inevitable, and appropriate interventions can reduce the impact of these problems. Following the emergence and worldwide spread of COVID-19, the growing popularity of smartphone health apps may represent an opportunity to improve cancer care and management. These apps can be used to collect objective data about patients’ behavior and behavior monitoring, which could help patients change their behavior, promote self-monitoring of symptoms, and enhance patients’ sense of empowerment and willingness to care for themselves [9] while allowing them to communicate with their health care team from a distance [10,11].

Various randomized controlled trials (RCTs) have found that mobile health (mHealth) interventions may be effective for adult cancer survivors. For example, mHealth interventions have increased the number of women screened for breast cancer [12]. Similarly, among patients with pancreatic ductal adenocarcinoma receiving chemotherapy, a mobile app intervention provided adequate nutritional and psychological support [13]. In addition, a web-based exercise intervention successfully increased the number of patients with cancer who engaged in physical activity [14]. Okunade et al [15] also predicted that telemedicine would be integrated into the care of patients in oncology following the COVID-19 pandemic; however, sufficient evidence to guide such integration has not been established. Owing to the issue of patients’ access, or lack thereof, to app interventions, it is difficult to design and implement unbiased, blinded RCTs to determine their true effects. The evidence for the efficacy of mHealth app interventions in cancer treatment might be unreliable. Some studies have demonstrated that smartphone app interventions benefit mental health [16,17]. By contrast, other studies have found no association between smartphone app interventions and psychological outcomes [18,19]. Further studies have suggested that apps increase patient anxiety and depression by enriching cancer information, which reminds them of what they are experiencing [20]. Thus, given this contradictory evidence, clarifying the psychological effects of app interventions remains difficult.

Although several systematic reviews have addressed the psychological impact of teleinterventions on cancer survivors [21-24], contradictory results remain. A meta-analysis that included 20 telehealth interventions found that the interventions improved patients’ QOL and self-efficacy and reduced depression, distress, and perceived stress. However, the interventions did not have any significant effect on anxiety [21]. Similarly, another meta-analysis of 14 phone-based interventions found that these interventions reduced anxiety and improved QOL but did not have any significant effect on depression [24]. No meta-analysis has comprehensively and specifically assessed the impact of smartphone apps on QOL and psychological symptoms in cancer survivors. Smartphone apps have natural advantages over websites and SMS text messaging, such as personalized design, rich mobile device features based on smartphones (cameras, phones, GPS, and contact lists), and timely push features. Therefore, smartphone app interventions may have higher adherence.

### Objectives

We conducted a systematic review and meta-analysis of RCTs to determine the effects of app interventions on QOL and psychological outcomes in adult cancer survivors. We also performed various subgroup analyses according to intervention duration, type of cancer, intervention theory, treatment strategy, and intervention delivery format to investigate the effects of app interventions.

## Methods

The meta-analysis adhered to the Cochrane Handbook guidelines for conducting systematic reviews and meta-analyses and the PRISMA (Preferred Reporting Items for Systematic Reviews and Meta-Analyses) guidelines and was registered in PROSPERO (International Prospective Register of Systematic Reviews; CRD42022370599).

### Ethical Considerations

This review did not require informed consent or ethics approval as the data were obtained from previously published studies.

### Article Selection and Search Strategy

We searched the following databases from inception to October 30, 2022: PubMed, Web of Science, Embase, the Cochrane Library, Scopus, China National Knowledge Infrastructure, and WanFang. For the literature search, we combined Medical Subject Headings and non–Medical Subject Heading terms, including (“cancer” OR “tumor” OR “neoplasms” OR “neoplasia”) AND (“mHealth applications” OR “mHealth” OR “portable software application” OR “app” OR “apps” OR “app-based” OR “electronic”) AND (“randomized controlled trial” OR “controlled clinical trial” OR “randomized” OR “placebo” OR “clinical trials as topic” OR “randomly” OR “trial”) NOT (“animals”) NOT (“humans” AND “animals”). There were no language restrictions. Additional relevant studies were identified by manually searching the references of the screened articles and reviews (Multimedia Appendix 1).

### Inclusion and Exclusion Criteria

The following criteria were used to determine whether to include each study: (1) adults with cancer (of any type or stage); (2) telehealth or telemedicine interventions delivered via an mHealth app; (3) a control group involving routine care, including usual care, waitlist control, conventional care, or health education delivered without the use of an mHealth app; (4) the outcome being QOL and psychological outcomes (including depression, anxiety, distress, and self-efficacy) with no restrictions on the measurement tools used; and (5) RCT study design. We excluded studies that used websites, SMS text messaging, email, or other technological interventions that did not include mHealth apps and studies that used mHealth apps without involving patients with cancer (eg, health care professionals who used mHealth apps). In addition, we excluded study protocols, reviews, and studies lacking complete data. The publication date was not restricted in any way.

### Data Extraction and Risk-of-Bias Assessment

The data management software EndNote X9 (Clarivate Analytics) was used. In total, 2 researchers (QMH and CB) independently extracted the data based on the qualifying criteria. Disagreements were resolved through discussion between the evaluators. If the data were duplicated or shared between studies, the most recently published or more comprehensive study was used in the analysis. We extracted the following data from each included study: first author, publication date, country, intervention theory, sample size, participant characteristics (mean age, type of cancer, and stage of cancer), intervention duration, treatment strategy, format of intervention delivery, and outcome measurements. The Cochrane risk-of-bias assessment tool was used to determine the risk of bias (Multimedia Appendix 2).

### Statistical Analysis

Following data extraction from the publications, heterogeneity tests and statistical analyses were conducted using RevMan (version 5.3; The Cochrane Collaboration) and Stata (version 16.0; StataCorp) software. As these included studies used various measuring tools, the standardized mean difference (SMD) with a 95% CI was used to estimate intervention effects on QOL, depression, anxiety, distress, and self-efficacy. If SDs were not provided, they were calculated using the available data. A 2-sided *P*<.05 was used to indicate a statistically significant difference in the overall effect. To determine the statistical heterogeneity of the included studies, the *I*^2^ statistic and *P* value were used. A fixed-effects model was used to pool the results if *I*^2^≤50% and *P*>.10; if heterogeneity was significant (*P*<.10 and *I*^2^>50%), a random-effects model was used to pool the results. If necessary and feasible, subgroup and sensitivity analyses were conducted to identify possible sources of between-study heterogeneity. Subgroup analyses were conducted based on intervention duration, type of cancer, intervention theory, treatment strategy, and intervention delivery format. Sensitivity analyses were carried out by omitting 3% (1/30) of the studies and modifying the pooling model (random-effects or fixed-effects models). To assess publication bias, the Begg and Egger regression tests were used.

## Results

### Characteristics of the Included Studies and Risk of Bias

**Figure 1:**
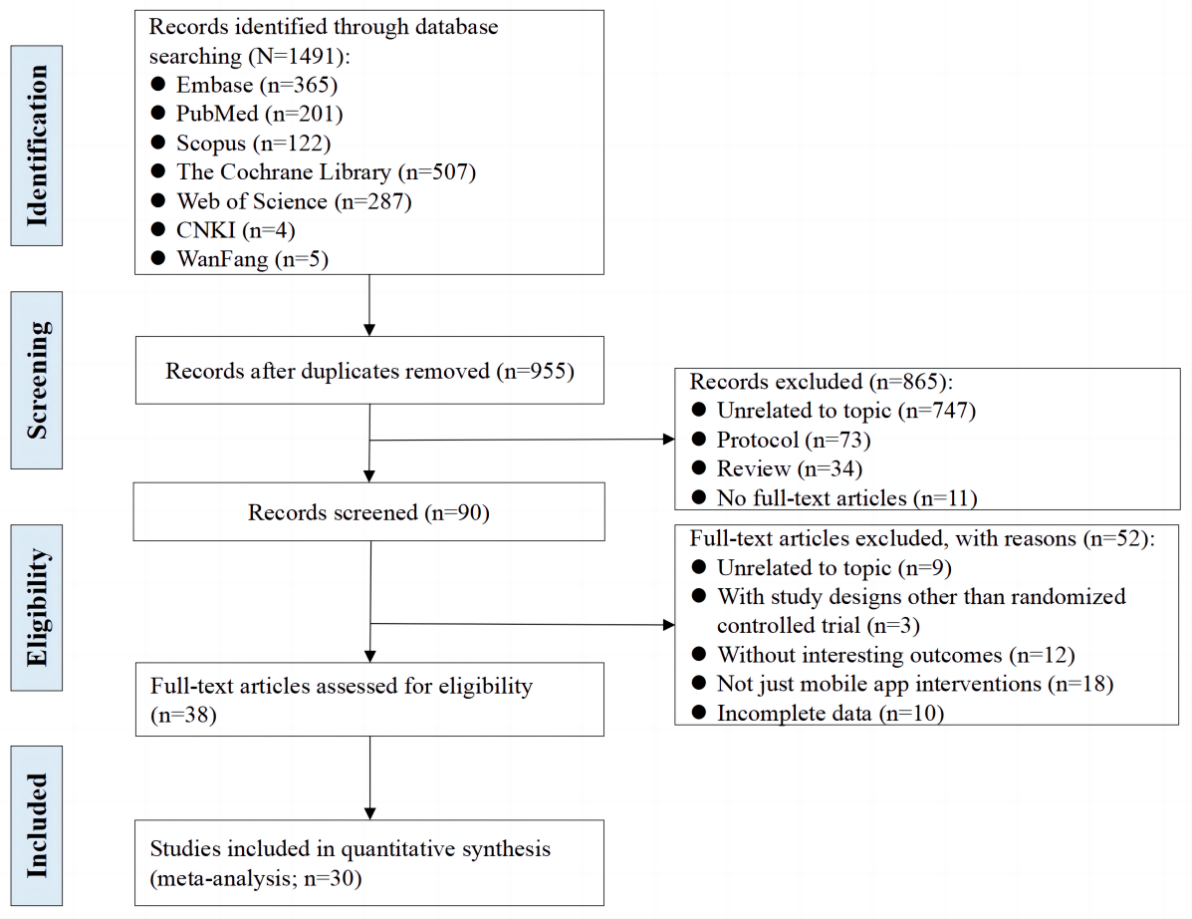
PRISMA (Preferred Reporting Items for Systematic Reviews and Meta-Analyses) flow diagram of study selection. CNKI: China National Knowledge Infrastructure.

The PRISMA flowchart depicts the extensive search process (Figure 1). Initially, 1491 articles were identified, with 38 (2.55%) records being further evaluated as potentially eligible. Finally, the meta-analysis included 2.01% (30/1491) of RCTs (with 5353 participants). Table 1 summarizes the characteristics of these studies. Each included study had a sample size ranging from 38 to 829. Participants’ age ranged from 41.9 (SD 11.30) to 67.1 (SD 10.4) years on average. The interventions lasted from 1 week to 12 months, with a median follow-up time of 2.8 months. Of the 30 studies, 13 (43%) included only patients with breast cancer; 7 (23%) used cognitive behavioral therapy interventions; and 9 (30%) and 7 (23%) included only patients treated with surgery and chemotherapy, respectively. In addition, different scales were used to assess the outcomes.

**Figure 2:**
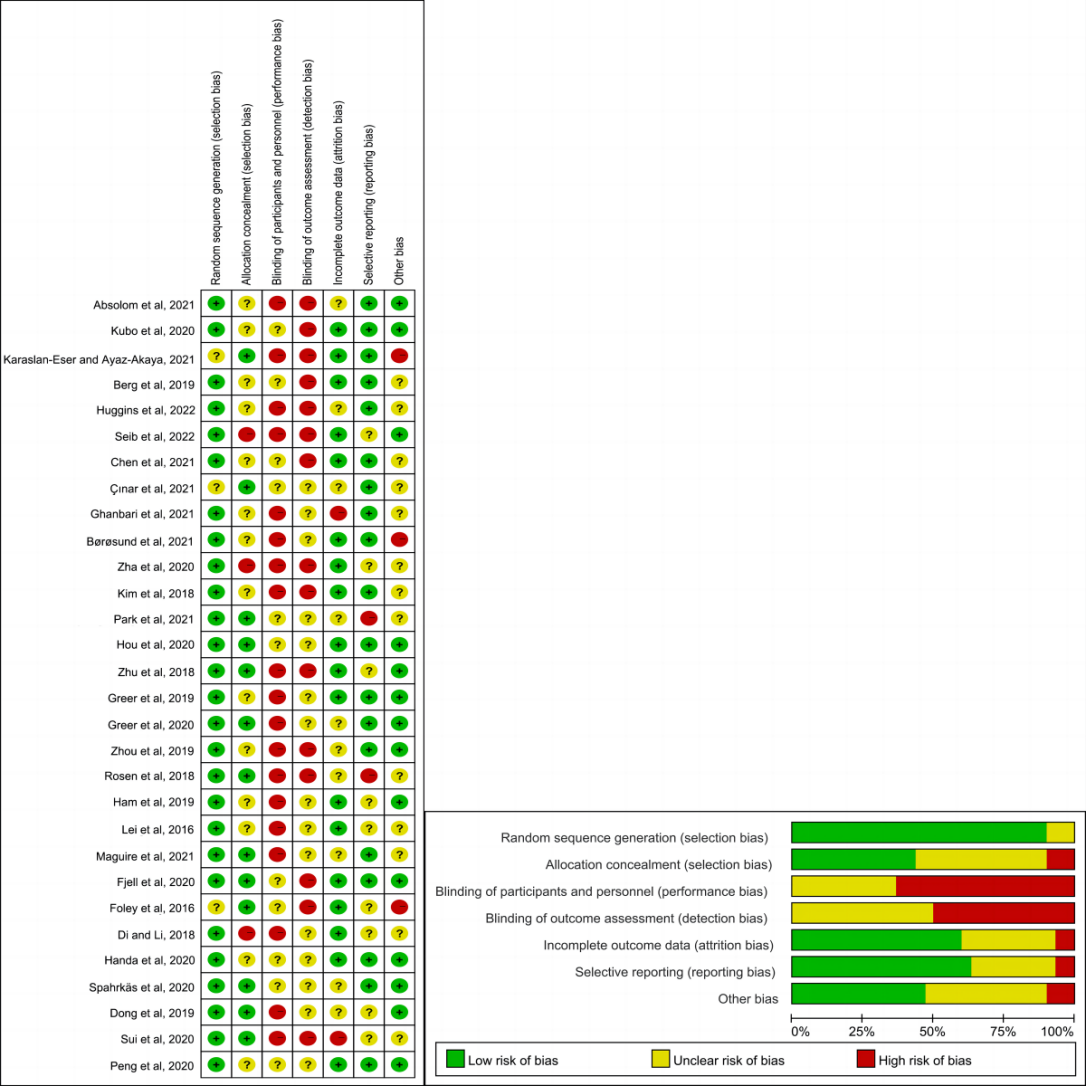
Risk-of-bias summary and graph [16-20,25-49].

The assessment of the risk of bias is shown in in Figure 2 [16-20,25-49] and Multimedia Appendix 2 [16-20,25-49]. The process of random sequence generation was explicitly described in 90% (27/30) of the studies. In 43% (13/30) of the studies, allocation concealment was adequately reported. A total of 63% (19/30) of the studies had a high risk of bias because of patients’ access or lack thereof to the mHealth app interventions, which made it difficult to blind participants and researchers. Regarding attrition bias, 33% (10/30) of the studies were rated as having an unclear risk of bias because of insufficient information on attrition. In comparison, 7% (2/30) of the studies were rated as having a high risk of bias because of high attrition rates. In total, 47% (14/30) of the studies published study protocols and reported all prespecified outcomes and were rated as having a low risk of reporting bias.

**Table 1:** Characteristics of the randomized controlled trial studies (N=30).

Author, year, and country	Intervention theory	Sample size, N		Patient characteristics						Intervention		Format of intervention delivery		Intervention duration	Outcomes and outcome measures
		Intervention	Control	Age (years), intervention	Age (years), control	Type of cancer	Stage of cancer	Treatment category							
Børøsund et al [25], 2021, Northern Europe	Cognitive behavioral theory	84	88	Mean 51.7 (SD 10.5)	Mean 52.3 (SD 12.0)	Breast cancer, brain cancer, prostate cancer, and others	Not restricted	Surgery, chemotherapy, radiation, and immune therapy	Intervention group: StressProffen app; control group: usual care		Interactive format (smartphone–based 2-way communication)		12 months		Anxiety (HADS-A^a^), depression (HADS-D^b^), and HRQOL^c^ (SF-36^d^)
Çınar et al [26], 2021, Turkey	Evidence-based symptom care theory	31	33	Mean 45.9 (SD 8.3)	Mean 45.5 (SD 9.8)	Breast cancer	Stage I to III	Surgery	Intervention group: mHealth^e^ app–based patient education; control group: routine care		Interactive format (smartphone–based 2-way communication)		12 months		QOL^f^ (FACT-ES QLS^g^) and distress (NCCN-DT^h^)
Fjell et al [27], 2020, Sweden	Unclear	74	75	Mean 48.0 (SD 10.6)	Mean 50.0 (SD 11.6)	Breast cancer	Not restricted	Chemotherapy	Intervention group: Interaktor app; control group: standard care		Interactive format (smartphone–based 2-way communication)		18 weeks		Distress (MSAS-GDI^i^) and QOL (EORTC QLQ-C30^j^)
Foley et al [20], 2016, Ireland	Unclear	13	26	Median 54 (IQR 49.5-61.5)	Median 52 (IQR 44-64)	Breast cancer	Not restricted	Surgery	Intervention group: Apple iPad; control group: standard care information		Didactic format (smartphone–based 1-way communication)		1 week		Anxiety (HADS-A) and depression (HADS-D)
Ghanbari et al [17], 2021, Iran	Cognitive behavioral theory	41	41	Mean 46.9 (SD 9.83)	Mean 46.0 (SD 8.80)	Nonmetastatic breast cancer	Not restricted	Not restricted	Intervention group: BCSzone app; control group: waitlist control		Interactive format (smartphone–based 2-way communication)		5 weeks		Anxiety (STAI^k^)
Greer et al [28], 2019, United States	Cognitive behavioral theory	72	73	Mean 55.86 (SD 10.08)	Mean 57.03 (SD 12.42)	Gastrointestinal cancer, gynecological cancer, lung cancer, breast cancer, and others	Stage IV or metastatic disease	Surgery, chemotherapy, radiation, and immune therapy	Intervention group: CBT^l^ mHealth app; control group: health education control		Interactive format (smartphone–based 2-way communication)		3 months		Anxiety (HADS-A), depression (HADS-D), and QOL (PHQ-9^m^)
Greer et al [29], 2020, United States	Unclear	91	90	Mean 52.85 (SD 13.74)	Mean 53.76 (SD 12.08)	Hematologic cancer, non–small cell lung cancer, breast cancer, high-grade glioma, sarcoma, and others	Not restricted	Chemotherapy	Intervention group: mHealth app; control group: standard care		Didactic format (smartphone–based 1-way communication)		3 months		QOL (FACT-G^n^)
Ham et al [30], 2019, Korea	CBT	21	21	Mean 41.90 (SD 11.30)	Mean 47.10 (SD 11.19)	Breast cancer, gynecological cancer, thyroid cancer, sarcoma, and others	Stage 0 to III	Surgery, radiotherapy, chemotherapy, and other treatments	Intervention group: HARUToday app; control group: waitlist control		Didactic format (smartphone–based 1-way communication)		10 weeks		Depression (BDI-II^o^), QOL (SF-36), and anxiety (STAI)
Handa et al [18], 2020, Japan	Unclear	47	48	Mean 49.9 (SD 0.2)	Mean 49.9 (SD 9.2)	Breast cancer	Not restricted	Chemotherapy	Intervention group: BPSS app; control group: ordinary instructions		Didactic format (smartphone–based 1-way communication)		12 weeks		Anxiety (HADS-A) and depression (HADS-D)
Karaaslan-Eser and Ayaz-Alkaya [31], 2021, Turkey	Unclear	42	42	Mean 60.33 (SD 9.31)	Mean 62.14 (SD 9.97)	Colorectal cancer, gastrointestinal stromal tumor, lung cancer, renal cell carcinoma, hepatocellular carcinoma, cholangiocarcinoma, and breast cancer	Stage III to IV	Oral anticancer agents	Intervention group: OKTED app; control group: standard care		Interactive format (smartphone–based 2-way communication)		6 months		Distress (MSAS-GDI)
Kim et al [19], 2018, Korea	Unclear	36	40	Median 49.8	Median 52.1	Breast cancer	Stage IV	Chemotherapy	Intervention group: mHealth game app; control group: conventional education		Interactive format (smartphone–based 2-way communication)		3 weeks		QOL (WHOQOL-BREF^p^ questionnaire), anxiety (STAI), and depression (BDI^q^)
Kubo et al [16], 2020, United States	Mindfulness-based therapy	31	46	Mean 65.8 (SD 8.8)	Mean 67.1 (SD 10.4)	Breast, hematological, gastrointestinal, lung, urological, and gynecological cancer	Not restricted	Not restricted	Intervention group: Headspace app; control group: waitlist control		Interactive format (smartphone–based 2-way communication)		3 months		Anxiety (HADS-A), depression (HADS-D), QOL (FACIT-Pal^r^), and distress (NCCN-DT)
Park et al [32], 2021, South Korea	Unclear	31	30	Mean 52.07 (SD 9.34)	Mean 54.74 (SD 7.87)	Breast cancer	Stage 0 to III	Surgery	Intervention group: Pillsy mHealth app; control group: usual care		Didactic format (smartphone–based 1-way communication)		4 weeks		Depression (Center for Epidemiologic Studies Depression Scale) and self-efficacy (General Self-Efficacy Scale)
Peng et al [33], 2020, China	Unclear	152	150	Mean 55.6 (SD 6.8)	Mean 56.3 (SD 7.0)	Not restricted	Not restricted	Not restricted	Intervention group: WeChat app; control group: usual care		Interactive format (smartphone–based 2-way communication)		3 days		QOL (cancer-related quality of life), anxiety (GAD-7^s^), and depression (PHQ-9)
Spahrkäs et al [34]; 2020, Australia, Canada, United Kingdom, and United States	CBT	519	280	Mean 56.7 (SD 9.99)	Mean 56.2 (SD 9.42)	Breast cancer, hematological cancer, digestive organ cancer, and others	Not restricted	Surgery, radiation therapy, chemotherapy, immunotherapy, stem cell transplant, hormone therapy, and other treatments	Intervention group: Untire mHealth app; control group: waiting list		Didactic format (smartphone–based 1-way communication)		3 months		QOL (EORTC QLQ-C30)
Sui et al [35], 2020, China	Unclear	100	100	Mean 61.37 (SD 11.21)	Mean 62.35 (SD 9.98)	Non–small cell lung cancer	Stage I to III	Surgery	Intervention group: WeChat app; control group: simple education and rehabilitation guidance		Interactive format (smartphone–based 2-way communication)		12 months		QOL (QLQ-C30), anxiety (HADS-A), and depression (HADS-D)
Zhou et al [36], 2019, China	Roy Adaptation Model	66	66	Mean 44.62 (SD 7.89)	Mean 44.37 (SD 7.32)	Breast cancer	Stage I to III	Surgery	Intervention group: CAT^t^+routine care; control group: routine care		Interactive format (smartphone–based 2-way communication)		3 months		Anxiety (SAS^u^) and depression (SDS^v^)
Zhu et al [37], 2018, China	The Bandura self-efficacy theory and the social exchange theory	57	57	Mean 46.2 (SD 8.5)	Mean 48.2 (SD 8.1)	Breast cancer	Stage 0 to III	Chemotherapy	Intervention group: BCS^w^+CAU^x^; control group: CAU		Interactive format (smartphone–based 2-way communication)		6 months		QOL (FACT-B^y^), anxiety (HADS-A), depression (HADS-D), self-efficacy (SICPA^z^), and distress (MDASI^a^^a^)
Di and Li [38], 2018, China	Unclear	65	67	Mean 44.32 (SD 11.03)	Mean 42.28 (SD 10.37)	Nasopharyngeal carcinoma	Stage 0 to IV	Radiotherapy and chemotherapy	Intervention group: smartphone medical app; control group: conventional follow-up visit		Interactive format (smartphone–based 2-way communication)		6 months		QOL (QLQ-C30)
Dong et al [39], 2019, China	Unclear	26	24	Mean 48.00 (SD 5.54)	Mean 51.63 (SD 7.49)	Breast cancer	Stage I to III	Surgery	Intervention group: social media apps; control group: traditional treatment and rehabilitation		Interactive format (smartphone–based 2-way communication)		3 months		QOL (SF-36)
Hou et al [40], 2020, China	Unclear	53	59	N/A^a^^b^	N/A	Breast cancer	Stage 0 to III	Not restricted	Intervention group: BCSMS^a^^c^ app + health care; control group: health care		Didactic format (smartphone–based 1-way communication)		3 months		QOL (QLQ-C30)
Lei [41], 2016, China	Orem self-care theory	58	58	N/A	N/A	Laryngeal cancer	Stage 0 to IV	Surgery	Intervention group: Rehab assistant app; control group: usual care		Interactive format (smartphone–based 2-way communication)		3 months		QOL (QLQ-C30)
Rosen et al [42], 2018, United States	Mindfulness training	57	55	Mean 51.40 (SD 10.73)	Mean 53.22 (SD 9.91)	Breast cancer	Stage 0 to IV	Not restricted	Intervention group: app-delivered mindfulness training; control group: waitlist control		Didactic format (smartphone–based 1-way communication)		3 months		QOL (FACT-B)
Zha [43], 2020, China	Unclear	41	41	Mean 45.14 (SD 11.14)	Mean 46.38 (SD 11.57)	Breast cancer	Stage I to II	Surgery	Intervention group: WeChat app care; control group: routine care		Interactive format (smartphone–based 2-way communication)		3 months		QOL (SF-36) and anxiety (STAI)
Absolom et al [44], 2021, United Kingdom	Unclear	256	252	Mean 55.9 (SD 12.2)	Mean 56.0 (SD 11.3)	Breast cancer, colon cancer, and gynecological cancer	Primary or local disease, metastatic	Chemotherapy	Intervention group: eRAPID; control group: routine care		Interactive format (mobile device–based 2-way communication)		18 weeks		QOL (FACT-G) and self-efficacy (Self-Efficacy Scale)
Berg et al [45], 2019, United States	Unclear	38	18	Mean 32.63 (SD 5.87)	Mean 32.39 (SD 4.60)	Breast cancer, lymphoma, and others	Stage 0 to IV	Not restricted	Intervention group: AWAKE; control group: attention control		Interactive format (mobile device–based 2-way communication)		6 months		QOL (QLQ-C30), depression (HADS-D), and self-efficacy (Self-Efficacy Scale)
Chen et al [46], 2021, China	Unclear	40	40	Mean 59.6 (SD 6.5)	Mean 59.8 (SD 7.0)	Esophageal cancer	Stage I to IIIa	Surgery	Intervention group: WeChat; control group: routine care		Interactive format (WeChat group–based 2-way communication)		3 months		QOL (QLQ-C30)
Huggins et al [47], 2022, Australia	CBT	36	37	Mean 66.6 (SD 9.7)	Mean 63.2 (SD 9.9)	Nasopharyngeal carcinoma	Not restricted	Not restricted	Intervention group: myPace; control group: routine care		Interactive format (app-based 2-way communication)		12 months		QOL (QLQ-C30)
Maguire et al [48], 2021, United Kingdom	Unclear	415	414	Mean 51.9 (SD 12.4)	Mean 52.9 (SD 12.1)	Breast cancer and colon cancer	Not restricted	Chemotherapy	Intervention group: ASyMS; control group: standard care		Didactic format (smartphone–based 1-way communication)		12 weeks		QOL (QLQ-C30), self-efficacy (CASE-cancer^a^^d^), and anxiety (STAI)
Seib et al [49], 2022, Australia	CBT	175	176	Mean 52.6 (SD 9.4)	Mean 53.7 (SD 8.1)	Breast cancer, gynecological cancer, and blood cancer	Not restricted	Not restricted	Intervention group: WWACP; control group: standard care		Interactive format (app-based 2-way communication)		12 weeks		QOL (SF-36)

^a^HADS-A: Hospital Anxiety and Depression Scale-Anxiety subscale.

^b^HADS-D: Hospital Anxiety and Depression Scale-Depression subscale.

^c^HRQOL: health-related quality of life.

^d^SF-36: 36-item Short Form Health Survey.

^e^mHealth: mobile health.

^f^QOL: quality of life.

^g^FACT-ES QLS: Functional Assessment of Cancer Therapy-Endocrine Symptoms Quality of Life Scale.

^h^NCCN-DT: National Comprehensive Cancer Network Distress Thermometer.

^i^MSAS-GDI: Memorial Symptom Assessment Scale-General Distress Index.

^j^EORTC QLQ-C30: European Organisation for Research and Treatment of Cancer Quality of Life Questionnaire C30.

^k^STAI: State-Trait Anxiety Inventory.

^l^CBT: cognitive behavioral therapy.

^m^PHQ-9: Patient Health Questionnaire-9.

^n^FACT-G: Functional Assessment of Cancer Therapy-General.

^o^BDI-II: Beck Depression Inventory-Second Edition.

^p^WHOQOL-BREF: World Health Organization Quality of Life-BREF questionnaire.

^q^BDI: Beck Depression Inventory.

^r^FACIT‐Pal: Functional Assessment of Chronic Illness Therapy-Palliative Care.

^s^GAD-7: Generalized Anxiety Disorder-7.

^t^CAT: cyclic adjustment training.

^u^SAS: Self-Rating Anxiety Scale.

^v^SDS: Self-Rating Depression Scale.

^w^BCS: breast cancer e-support.

^x^CAU: care as usual.

^y^FACT-B: Functional Assessment of Cancer Therapy-B.

^z^SICPA: Stanford Inventory of Cancer Patient Adjustment.

^aa^MDASI: MD Anderson Symptom Inventory.

^ab^N/A: not applicable.

^ac^BCSMS: Breast Cancer Self-Management Support.

^ad^CASE-cancer: Communication and Attitudinal Self-Efficacy scale for cancer.

### Functions of Smartphone Apps

The functions of these apps can be classified as follows: provision of health education and advice, physician-patient communication via the mHealth app, and data management regarding self-management behaviors of patients with cancer (including data upload, visualization, and reminder services). Physicians and patients interact in 2 ways: the app generates automated feedback based on predesigned personalized feedback, and medical professionals issue interactive guidance based on patient-provided personalized data. Most (22/30, 73%) of these studies incorporated personalized guidance services provided by health care professionals who analyzed patient data and communicated with the patients via SMS text message, phone, or video.

### Effects on QOL

**Figure 3:**
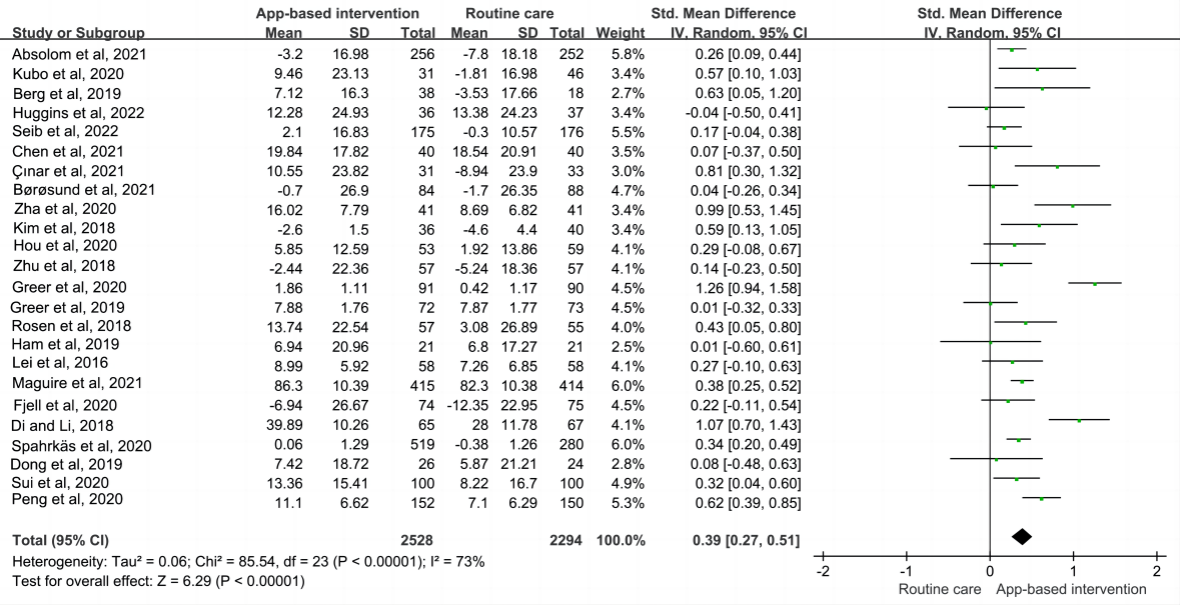
Meta-analysis on quality of life [16,19,25-46]. IV: inverse variance; Std: standardized.

A total of 80% (24/30) of the studies [16,19,25-30,33-35,37-49] involving 4822 participants used various scales to report the outcome of QOL. Of these 24 studies, 8 (33%) [19,26,27,37,39,40,42,43] focused on patients with breast cancer, and the other 16 (67%) included patients with multiple types of cancer (such as breast cancer, brain cancer, and prostate cancer). A total of 62% (15/24) of the studies had an intervention duration of <3 months, and the remainder had an intervention duration of 3 to 12 months. The apps used different intervention theories (including cognitive behavioral therapy, psychoeducation, and mindfulness-based stress reduction); 25% (6/24) of the studies used cognitive behavioral therapy interventions, and 8% (2/24) of the studies were based on mindfulness-based therapy. In these studies, patients with cancer received different treatment strategies; 29% (7/24) of the studies were conducted only among patients under chemotherapy, and 25% (6/24) were conducted only in patients undergoing surgery. Owing to the significant heterogeneity among the studies (*P*<.001; *I*^2^=77%), the results were pooled using a random-effects model. Overall, the mHealth app interventions significantly improved cancer-related QOL scores (SMD=0.39, 95% CI 0.27-0.51; *P*<.001; Figure 3 [16,19,25-30,33-35,37-49]).

We conducted subgroup analyses according to intervention duration, type of cancer, intervention theory, treatment strategy, and intervention delivery format to investigate potential sources of heterogeneity. Pooled results for the short-term (≤3 months) follow-up period suggested that mHealth app medical interventions were effective in improving QOL (SMD_<3 months_=0.41, 95% CI 0.26-0.57; *P*=.001; SMD_3 to 12 months_=0.36, 95% CI 0.14-0.57; *P*=.001; Table 2 and Multimedia Appendix 3 [16,19,25-30,33-35,37-49]). When studies were grouped by type of cancer, the results showed that mHealth app interventions may improve cancer-related QOL scores across cancer types (SMD_Breast cancer_=0.42, 95% CI 0.21-0.63; *P*<.001; SMD_Various cancers_=0.38, 95% CI 0.23-0.53; *P*=.001; Table 2 and Multimedia Appendix 4 [16,19,25-30,33-35,37-49]). Subgroup analyses of different intervention theories revealed low heterogeneity for cognitive behavioral theory (35%) and mindfulness-based theory (0%), implying that different intervention theories may be an important source of heterogeneity (Table 2 and Multimedia Appendix 5 [16,19,25-30,33-35,37-49]). Studies grouped by intervention delivery format revealed that these interventions significantly improved cancer-related QOL scores across different intervention delivery formats (SMD_Interactive format_=0.36, 95% CI 0.22-0.50; *P*<.001; SMD_Didactic format_=0.48, 95% CI 0.22-0.73; *P*<.001; Table 2 and Multimedia Appendix 6 [16,19,25-30,33-35,37-49]). There were no significant differences in QOL scores, but there was a high heterogeneity among patients with cancer receiving different treatment modalities (Table 2 and Multimedia Appendix 7 [16,19,25-30,33-35,37-49]).

In the sensitivity analysis, switching from a random-effects model to a fixed-effects model confirmed the effect of the app interventions (SMD=0.43, 95% CI 0.35-0.50; *P*<.001). Furthermore, when each study was excluded sequentially, the pooled estimates remained robust, ranging from 0.38 (95% CI 0.30-0.45) to 0.46 (95% CI 0.37-0.54). There was no evidence of publication bias (Begg test: *P*=.65; Egger test: *P*=.67; Multimedia Appendix 8). Therefore, the pooled estimate for QOL was robust.

**Table 2:** Subgroup analyses of quality of life (N=24).

Stratification			Studies, n (%)		*P* value for heterogeneity		*I*^2^ (%)		Pooled standardized mean difference (95% CI)		*P* value for pooled results
**Intervention duration (months)**											
	<3	15 (62)		<.001		75		0.41 (0.26-0.57)		.001^a^	
	3 to 12	9 (38)		<.001		72		0.36 (0.14-0.57)		.001^a^	
**Types of cancer**											
	Breast cancer	8 (33)		.05		51		0.42 (0.21-0.63)		.001^a^	
	Various cancers	16 (67)		<.001		79		0.38 (0.23-0.53)		.001^a^	
**Intervention theory**											
	Cognitive behavioral theory	6 (25)		.17		35		0.16 (0.01-0.30)		.03^a^	
	Mindfulness-based theory	2 (8)		.65		0		0.48 (0.19-0.77)		.01^a^	
	Other theories	16 (67)		<.001		76		0.49 (0.33-0.66)		.001^a^	
**Format of intervention delivery**											
	Interactive format (smartphone–based 2-way communication)	18 (75)		<.001		68%		0.36 (0.22-0.50)		<.001^a^	
	Didactic format (smartphone–based 1-way communication)	6 (25)		<.001		83%		0.48 (0.22-0.73)		<.001^a^	
**Treatment category**											
	Patients for chemotherapy	7 (29)		<.001		87%		0.55 (0.27-0.82)		<.001^a^	
	Patients for surgery	6 (25)		.02		62%		0.41 (0.13-0.69)		.004^a^	
	Patients for various treatments	11 (46)		.02		53%		0.28 (0.14-0.42)		.007^a^	

^a^*P*<.05.

### Effects on Anxiety

**Figure 4:**
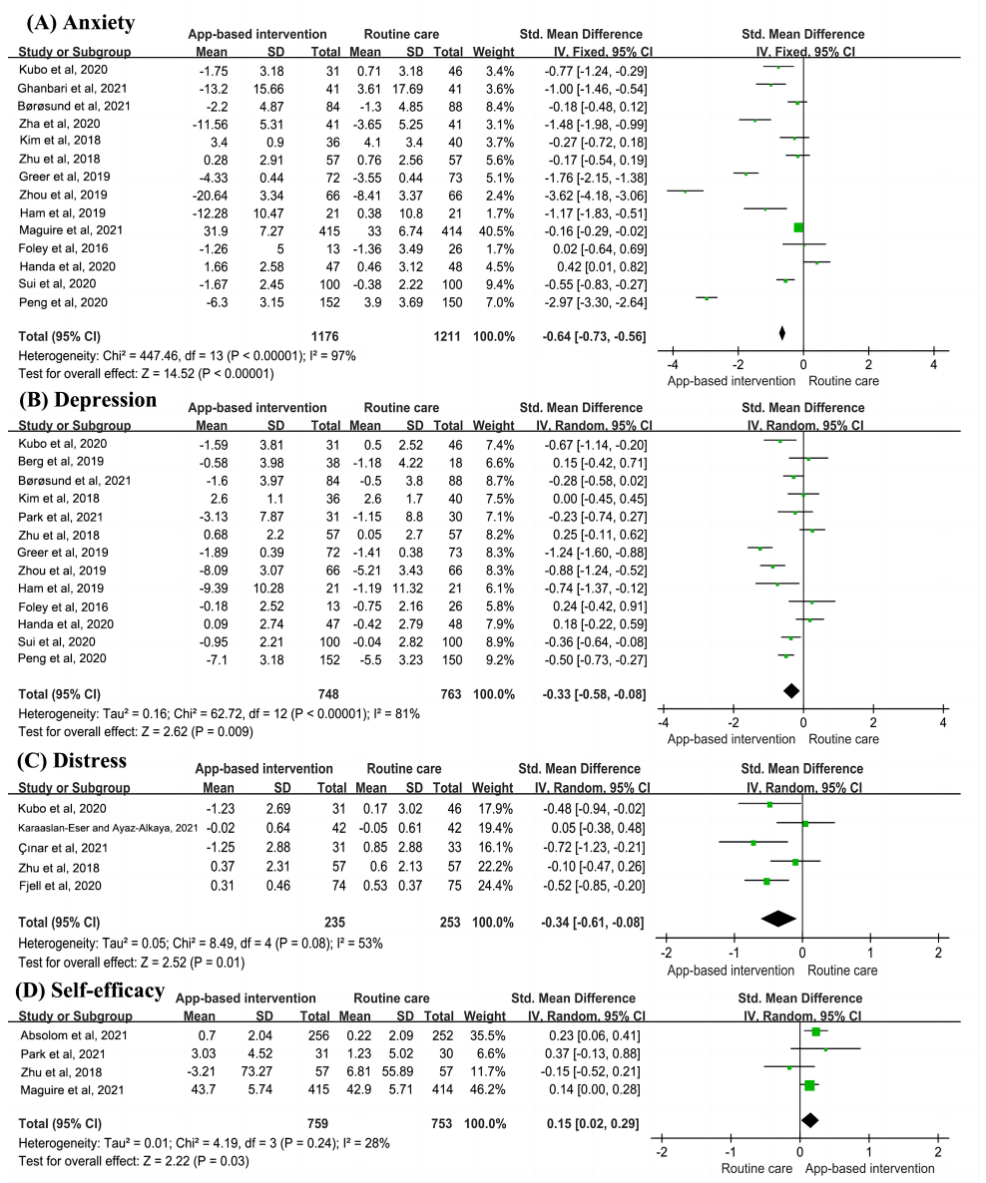
Meta-analysis on (A) anxiety, (B) depression, (C) distress, and (D) self-efficacy [16-20,25-28,30,31,33,34,40-42,45,47-49]. IV: inverse variance; Std: standardized.

A total of 47% (14/30) of the studies [16-20,25,28,30,33,35-37,43,48] measured anxiety scores using different scales. Overall, the mHealth app interventions significantly alleviated anxiety among cancer survivors, but there was high heterogeneity (SMD=−0.64, 95% CI −0.73 to −0.56; *P*<.001; *I*^2^=97%; Figure 4 [16-20, 25-28, 30-33, 35-37, 43-45, 48]).

On the basis of groups of intervention duration, 79% (11/14) of the studies had an intervention duration of <3 months, and 21% (3/14) had an intervention duration of 3 to 12 months. Subgroup analyses showed that these app-based interventions were still effective with different intervention durations. A total of 50% (7/14) of the studies [17-20,37,43,48] compared anxiety scores among breast cancer survivors and showed poor app intervention outcomes (SMD=−0.87, 95% CI −1.79 to 0.05; *P*=.06; *I*^2^=96%). Subgroup analyses by different intervention theories revealed high heterogeneity among interventions based on cognitive behavioral theory, but these were still effective in alleviating anxiety. Furthermore, subgroup analyses revealed that mHealth app interventions with an interactive format significantly reduced cancer-related anxiety scores (SMD=−1.27, 95% CI −1.99 to −0.56; *P*=.001; *I*^2^=97%). When studies were grouped by treatment strategy, app interventions did not alleviate anxiety in patients in chemotherapy (SMD=−0.06, 95% CI −0.32 to 0.19; *P*=.62; *I*^2^=60%) but could alleviate anxiety in patients undergoing surgery or comprehensive treatment (Table 3).

We found no significant change in the pooled estimates when single studies were excluded sequentially and the pooled model was changed. No evidence of publication bias was found (Begg test: *P*=.69; Egger test: *P*=.30; Multimedia Appendix 8). Therefore, the pooled estimate for anxiety was robust.

**Table 3:** Subgroup analyses of anxiety (N=14).

Stratification			Studies, n (%)		*P* value for heterogeneity		*I*^2^ (%)		Pooled standardized mean difference (95% CI)		*P* value for pooled results
**Intervention duration (months)**											
	<3	11 (79)		<.001		98		−1.16 (−1.91 to −0.41)		.002^a^	
	3 to 12	3 (21)		.14		49		−0.32 (−0.57 to −0.06)		.01^a^	
**Types of cancer**											
	Breast cancer	7 (50)		<.001		96		−0.87 (−1.79 to 0.05)		.06	
	Various cancers	7 (50)		<.001		97		−1.07 (−1.86 to −0.29)		.006^a^	
**Intervention theory**											
	Cognitive behavioral theory	4 (29)		<.001		93		−1.02 (−1.81 to −0.23)		.01^a^	
	Other theories	10 (71)		<.001		98		−0.95 (−1.68 to −0.23)		.01^a^	
**Format of intervention delivery**											
	Interactive format (smartphone–based 2-way communication)	10 (71)		<.001		97		−1.27 (−1.99 to −0.56)		.001^a^	
	Didactic format (smartphone–based 1-way communication)	3 (21)		<.001		82		−0.17 (−0.67 to 0.32)		.49	
**Treatment category**											
	Patients for chemotherapy	4 (29)		.06		60		−0.06 (−0.32 to 0.19)		.62	
	Patients for surgery	4 (29)		<.001		97		−1.41 (−2.81 to −0.01)		.04^a^	
	Patients for various treatments	6 (43)		<.001		97		−1.31 (−2.26 to −0.36)		.007^a^	

^a^*P*<.05.

### Effects on Depression

The meta-analysis for depression included 1511 patients from 43% (13/30) of the studies [16,18-20,25,28,30,32,33,35-37,45]. A random-effects model was chosen for analysis owing to the significant heterogeneity among the 43% (13/30) of the studies (*P*<.001; *I*^2^=81%). The pooled results indicated that the mHealth app intervention group had a lower depression score than the routine care group (SMD=−0.33, 95% CI −0.58 to −0.08; *P*=.009; Figure 4).

Grouping by intervention duration, 69% (9/13) of the studies had an intervention duration of <3 months, and 31% (4/13) had an intervention duration of 3 to 12 months. Subgroup analyses showed that these app-based interventions were effective with durations of <3 months but not with a duration of 3 to 12 months (SMD=−0.25, 95% CI −0.51 to 0.02; *P*=.07; *I*^2^=53%). When studies were grouped by type of cancer, 46% (6/13) of the studies involved breast cancer survivors [21-23,43,47,48], and mHealth app interventions did not alleviate depression in these survivors (SMD=−0.11, 95% CI −0.27 to 0.06; *P*=.21). Subgroup analyses according to intervention theory revealed that cognitive behavioral theory–based interventions could effectively relieve depression in cancer survivors (SMD=−0.75, 95% CI −1.42 to 0.09; *P*=.03), but there was high heterogeneity. A subgroup analysis revealed that mHealth app interventions with an interactive format significantly reduced cancer-related depression (SMD=−0.41, 95% CI −0.70 to −0.12; *P*=.006), but didactic format interventions were not effective in improving depression scores (SMD=−0.12, 95% CI −0.54 to 0.30; *P*=.58). When studies were grouped by treatment strategy, researchers found that app interventions did not alleviate depression in survivors who were treated with chemotherapy and surgery but could alleviate depression in survivors with comprehensive treatment (SMD=−0.56, 95% CI −0.90 to −0.21; *P*=.001; *I*^2^=79%; Table 4).

The fixed-effects model produced the same outcome as the random-effects model in the sensitivity analysis. In addition, when using a single-study approach, we found no studies that significantly altered the pooled results. No significant publication bias was found (Begg test: *P*=.58; Egger test: *P*=.49; Multimedia Appendix 8).

**Table 4:** Subgroup analyses of depression (N=13).

Stratification			Studies, n (%)		*P* value for heterogeneity		*I*^2^ (%)		Pooled standardized mean difference (95% CI)		*P* value for pooled results
**Intervention duration (months)**											
	<3	9 (69)		<.001		82		−0.45 (−0.77 to −0.13)		.006^a^	
	3 to 12	4 (31)		.09		53		−0.25 (−0.51 to 0.02)		.07	
**Types of cancer**											
	Breast cancer	7 (54)		<.001		77		−0.11 (−0.27 to 0.06)		.21	
	Various cancers	6 (46)		.001		75		−0.55 (−0.68 to −0.42)		.006^a^	
**Intervention theory**											
	Cognitive behavioral theory	3 (23)		<.001		88		−0.75 (−1.42 to −0.09)		.03^a^	
	Other theories	10 (77)		<.001		75		−0.21 (−0.46 to 0.04)		.10	
**Format of intervention delivery**											
	Interactive format (smartphone–based 2-way communication)	9 (69)		<.001		84		−0.41 (−0.70 to −0.12)		.006^a^	
	Didactic format (smartphone–based 1-way communication)	4 (31)		.07		58		−0.12 (−0.54 to 0.30)		.58	
**Treatment category**											
	Patients for chemotherapy	3 (23)		.69		0		0.16 (−0.07 to 0.40)		.17	
	Patients for surgery	4 (31)		.01		72		−0.37 (−0.77 to 0.03)		.07	
	Patients for various treatments	6 (46)		.002		79		−0.56 (−0.90 to −0.21)		.001^a^	

^a^*P*<.05.

### Effects on Distress

The meta-analysis of distress included 17% (5/30) of the studies [16,26,27,31,43] with a total of 488 cancer survivors. As there was heterogeneity among the studies (*P*=.08; *I*^2^=53%), a random-effects model was used to pool the results. Overall, the mHealth app interventions significantly alleviated distress among cancer survivors (SMD=−0.34, 95% CI −0.61 to −0.08; *P*=.01; Figure 4). To assess the robustness of the pooled results, we performed sensitivity analyses using various pooled models. The pooled results of the fixed-effects model also showed that the app intervention group had lower distress scores than the usual care group (SMD=−0.34, 95% CI −0.52 to −0.16; *P*=.006), indicating that the pooled effect size was robust. Publication bias was not examined as <10 studies were included.

### Effects on Self-efficacy

A total of 13% (4/30) of the studies [32,37,44,45] reported self-efficacy as an outcome. Pooling of studies showed a statistically significant effect size favoring the intervention group (SMD=0.15, 95% CI 0.02-0.29; *P*=.03; *I*^2^=28%). The fixed-effect model also showed that app interventions had higher self-efficacy scores than usual care (SMD=0.16, 95% CI 0.06-0.26; *P*=.008; Figure 4).

## Discussion

### Principal Findings

Currently, the medical pattern is changing from a biomedical pattern (the treatment of disease only focusing on the patient’s physical function) to a biopsychosocial medical pattern (the treatment of disease with comprehensive consideration of the patient’s physical function, mental health, and social environment). Thus, greater attention is being paid to patients’ mental health and social functioning. Among cancer survivors, symptoms such as depression, anxiety, distress, and pain are prevalent and undertreated, which may negatively affect their QOL and self-efficacy. However, smartphone users are increasing worldwide and are expected to reach 6.8 billion by 2023, with a smartphone penetration rate of 53.8% [50]. Furthermore, smartphone apps have natural advantages over websites, SMS text messages, and other similar communication methods owing to their personalized design, rich mobile device features (such as cameras, phones, GPS, and contact lists), and timely push features. Therefore, the use of smartphone health apps could be a potentially effective way to improve mental health and social functioning among patients with cancer.

We included 30 RCTs in this meta-analysis, and all studies (30/30, 100%) provided smartphone app interventions for cancer survivors. The pooled results showed that smartphone app–based interventions improved QOL (SMD=0.39; *P*<.001) and self-efficacy (SMD=0.15; *P*=.03) in cancer survivors compared with conventional care education and significantly reduced adverse psychological outcomes (anxiety, depression, and distress). In particular, short-term interventions (duration of ≤3 months), physician-patient interaction interventions (2-way communication using a smartphone app), and cognitive behavioral therapy–based interventions might be most effective for improving QOL and alleviating adverse psychological effects.

### Interpretation of Findings

The effect of mHealth app interventions on QOL, anxiety, depression, distress, and self-efficacy in adult cancer survivors over a median follow-up time of 2.8 months was consistent with recent results regarding cell phone, SMS text message, and web-based interventions [21,22,26]. This effect can be attributed to the prevalence and inherent advantages of smartphones. Compared with routine care, app-based interventions can provide more visually based and vivid educational counseling, enabling patients to establish close and ongoing contact with their treatment team [51,52]. Furthermore, with such an intervention, cancer survivors may become more aware of their condition and learn to cope with some of the problems associated with cancer [51]; as a result, patients may have a greater sense of empowerment and willingness to care for themselves, thereby improving their QOL and alleviating adverse psychological effects [53]. In addition, as a high financial burden is associated with a low QOL and high anxiety in cancer survivors [54], app interventions can help reduce health care costs, further improving patients’ QOL and alleviating adverse psychological effects [55].

In this review, we conducted subgroup analyses according to intervention duration, type of cancer, intervention theory, treatment category, and intervention delivery format. We found that the short-term effects of app interventions on QOL and psychological outcomes (median follow-up period of 2.8 months) were superior to the long-term effects, which were inconsistent for QOL, anxiety, and depression. This may be influenced by the progression, vulnerability, and persistence of cancer itself. However, this highlights the need for further research to test the effectiveness of mHealth interventions over the long term. Pooled results from studies on patients with breast cancer found that, although tending to alleviate anxiety and depression (SMD <0), app interventions did not significantly improve patients’ anxiety and depression status. In female patients, the rich cancer information within an app may remind them of what they are experiencing, leading to increased anxiety and depression [18]. Therefore, clinical practitioners should further explore appropriate care for patients with breast cancer based on evidence-based research and cognitive behavioral therapy. Among the different formats of intervention delivery, most (22/30, 73%) studies used app monitoring combined with feedback interventions, which significantly improved patients’ anxiety and depression. On the one hand, cancer survivors may become more aware of their condition through disease self-monitoring and learning to cope with some cancer-related problems [51]. By contrast, by conducting physician-patient communication via an app, patients with cancer may develop a close and ongoing partnership with their treatment team and communicate more effectively regarding disease progression or treatment complications. However, the effectiveness of educational message delivery may depend on how easily the patient understands the content and the importance of the message. Therefore, interventions in a didactic format to deliver educational messages have not been effective in alleviating anxiety and depression. Our review showed that cognitive behavioral therapy was effective in improving QOL and alleviating adverse psychological effects among cancer survivors. This result is consistent with those of other studies [56,57]. A possible explanation is that cognitive behavioral therapy interventions for patients address a broad range of aspects, such as physical, psychological, and social aspects, which can improve QOL and alleviate adverse psychological effects. However, relevant studies have been conducted among patients with cancer using an app, which cannot be compared directly with breast cancer treatment. Therefore, these results should be interpreted with caution.

The results of this meta-analysis indicated a significant improvement in QOL among adult cancer survivors who received chemotherapy. This was similar to the findings of 2 previous meta-analyses, which also found a significant improvement in QOL [21,24]. However, the intervention effects on anxiety and depression remain unclear as there was no significant difference between the intervention and control groups for both outcomes. A total of 2 previous meta-analyses regarding the effects of mobile phone–based interventions on anxiety and depression in this patient population also yielded contrasting results [21,24]. One study found that anxiety but not depression was significantly reduced [24], whereas the other study reported inverse findings [21]. These inconsistencies point to the need for further research to test the effectiveness of mHealth interventions on anxiety and depression in patients with cancer.

### Study Limitations

This study had some limitations. First, the included studies had qualitative and methodological weaknesses. Most studies failed to elucidate the processes of allocation concealment (17/30, 57%), researcher or participant blinding (19/30, 63%), and strategies for handling incomplete outcome data. Therefore, the design of allocation concealment, participant blinding, and outcome assessment should be emphasized in future studies to draw more credible conclusions. Second, there is a huge variation in the conceptualization and operationalization of patient participation, which makes data synthesis extremely difficult. The effects of app interventions should be interpreted with caution owing to the high heterogeneity in the operational definitions of measurement instruments and instrument scoring systems. However, this meta-analysis included only RCTs and used random-effects models to pool results when appropriate to yield the most conservative estimates. Subgroup and sensitivity analyses were also performed, and the results showed that the pooled estimates were relatively robust. In addition, because of the limitations of the included studies, we did not conduct subgroup analyses on the frequency of physician-patient interactions via apps; previous studies suggested that app interaction frequency leads to different effects [58]. Therefore, further studies should be conducted on interaction frequency. Finally, the extraction and classification of interventions is challenging because of considerable heterogeneity in the design of the interventions. The risk of misclassification of intervention characteristics and the exploratory nature of our subgroup analyses prevented us from drawing reliable conclusions about the characteristics of effective interventions.

### Implications

Our findings have several important implications. First, at a median follow-up time of 2.8 months, mobile app interventions may have a significant effect on enhancing QOL in cancer survivors and alleviating anxiety, depression, and distress in these patients. However, there is an urgent need to assess the long-term effects of these interventions on QOL and psychological outcomes. Second, using a physician-patient interaction intervention is more likely to significantly improve QOL and psychological effects. Future clinical research should further explore care modalities of patients with cancer based on the physician-patient interaction format. Third, cognitive behavioral therapy interventions address many aspects, such as physical, psychological, and social aspects, which improves QOL and alleviates adverse psychological effects. In the future, the development of mHealth apps that are based on cognitive behavioral theory should be encouraged. Fourth, clinical practitioners should further explore appropriate care strategies for breast cancer survivors. Fifth, it is difficult to identify patterns of patient engagement with smartphone app–based interventions because of the wide variability in intervention design and measurement tool scoring systems among the studies. By exploring factors such as participant characteristics and active engagement, further insights can be gained into strategies that can help increase patients’ motivation to participate and maintain intervention integrity.

### Conclusions

This review showed that smartphone app–based interventions might help address certain psychological issues experienced by cancer survivors. In particular, short-term interventions (duration of ≤3 months), physician-patient interaction interventions (2-way communication using a smartphone app), and cognitive behavioral therapy–based interventions might be more effective in improving QOL and alleviating adverse psychological effects. However, the evidence supporting these interventions is still being gathered and is not yet fully conclusive. Further rigorous and well-designed studies are warranted to address the methodological flaws identified in this review. In conclusion, mHealth interventions may be effective in providing psychological support for adult cancer survivors.

## Appendix

Multimedia Appendix 1 Searching strategy.

Multimedia Appendix 2 Risk of bias.

Multimedia Appendix 3 Pooled results for quality of life grouped by intervention duration. IV: inverse variance; Std: standardized.

Multimedia Appendix 4 Pooled results for quality of life grouped by type of cancer. IV: inverse variance; Std: standardized.

Multimedia Appendix 5 Pooled results for quality of life grouped by intervention theory. IV: inverse variance; Std: standardized.

Multimedia Appendix 6 Pooled results for quality of life grouped by format of intervention delivery. IV: inverse variance; Std: standardized.

Multimedia Appendix 7 Pooled results for quality of life grouped by treatment strategy. IV: inverse variance; Std: standardized.

Multimedia Appendix 8 Funnel plots of quality of life, anxiety, and depression. SMD: standardized mean difference.

## Abbreviations

mHealth: mobile health
PRISMA: Preferred Reporting Items for Systematic Reviews and Meta-Analyses
PROSPERO: International Prospective Register of Systematic Reviews
QOL: quality of life
RCT: randomized controlled trial
SMD: standardized mean difference
