# Supplementary material for: Effect of Mobile Phone App–Based Interventions on Quality of Life and Psychological Symptoms Among Adult Cancer Survivors: Systematic Review and Meta-analysis of Randomized Controlled Trials
Source: J Med Internet Res. 2022 Dec 19;24(12):e39799. doi: 10.2196/39799 (PMC9808609; doi:10.2196/39799)
Supplement: Multimedia Appendix 1 [file jmir_v24i12e39799_app1.docx]

**Table S1. Searching strategy**

|  | **Key words** | **English Synonyms** | **Emtree / MeSH/ Subject/** **Central**  **Controlled Vocabulary** |
| --- | --- | --- | --- |
| Population | Cancer | Neoplas*  Tumor*  Tumour*  Cancer*  Malignan*  Oncolog*  Leukemi*  Leukaemi*  Lymphoma*  Sarcoma*  Carcino* | Neoplasm  Oncology  Cancer patient  Neoplasms  Medical oncology  Cancer  Oncology  Cancer patients  肿瘤  癌 |
| Intervention | mobile applications | mobile app*  software app*  smartphone app*  portable electronic app*  mhealth*  mobile health app*  mhealth intervention*  mhealth app*  app  mobile intervention*  smartphone intervention*  smartphone app*  mobile phone app*  mobile phone intervention* | mobile application  smartphone application  mhealth  Telemedicine  移动应用  远程应用 |
| Outcome | Quality of life,  mental health (Anxiety,  Depression, distress, psychological Stresses, self efficacy) | Quality of life  Psychologi*  Mental*  Anxiet*  Anxious*  Depress*  Depressive*  Distress*  psychological stress*  self effica* | Quality of life  mental health  Anxiety  Anxiety disorders  Depression  Depressive disorders  Distress  Psychological distress  Mental disorders  Stress, psychological  self efficacy  心理问题  生活质量  焦虑  抑郁 |
| Study design | Randomized control trails | Random*  RCT | randomized controlled trial  controlled clinical trial  clinical trials as topic  Randomly  随机对照  临床试验 |

| Database |  | Search syntax | Citations found |
| --- | --- | --- | --- |
| 1) Embase | 1 | (“Neoplas*” OR “Tumor*” OR “Tumour*” OR “Cancer*” OR “Malignan*” OR “Oncolog*” OR “Leukemi*” OR “Leukaemi*” OR “Lymphoma*” OR “Sarcoma*” OR “Carcino*”):ti,ab,kw |  |
|  | 2 | “Neoplasm”/exp OR “Oncology”/exp OR “Cancer patient”/exp |  |
|  | 3 | (“mobile app*” OR “software app*” OR “smartphone app*” OR “portable electronic app*” OR “mhealth*” OR “mobile health app*” OR “mhealth intervention*” OR “mhealth app*” OR app OR “mobile intervention*” OR “smartphone intervention*” OR “smartphone app*” OR “mobile phone app*” OR “mobile phone intervention*”):ti,ab,kw |  |
|  | 4 | “mobile application”/exp OR “smartphone application”/exp OR “telemedicine”/exp OR “mhealth”/exp |  |
|  | 5 | (“Quality of life” OR “Psycholog*” OR “mental health” OR “Anxiet*” OR “Anxious*” OR “Depress*” OR “Depressive*” OR “Distress*” OR “psychological stresses” OR “self effica*”):ti,ab,kw |  |
|  | 6 | “Quality of life”/exp OR “mental health”/exp OR “psychological”/exp OR “Anxiety”/exp OR “Anxiety disorders”/exp OR “Depression”/exp OR “depression disorders”/exp OR “distress”/exp OR “psychological stresses”/exp OR “self efficacy”/exp |  |
|  | 7 | (#1 OR #2) AND (#3 OR #4) AND (#5 OR #6) |  |
|  | 8 | (#1 OR #2) AND (#3 OR #4) AND (#5 OR #6) AND [embase]/lim AND ('randomized controlled trial'/de OR 'randomized controlled trial topic'/de) AND ([adult]/lim OR [aged]/lim) AND [english]/lim | **365** |
| 2) PubMed | 1 | Neoplas*[tw] OR Tumor*[tw] OR Tumour*[tw] OR Cancer*[tw] OR Malignan* [tw] OR Oncolog*[tw] OR Leukemi*[tw] OR Leukaemi*[tw] OR Lymphoma* OR Sarcoma*[tw] OR Carcino*[tw] OR adenocarcinoma*[tw] |  |
|  | 2 | “Neoplasms”[mh] OR “Medical oncology”[mh] |  |
|  | 3 | “mobile app*”[tw] OR “software app*”[tw] OR “smartphone app*”[tw] OR “portable electronic app*”[tw] OR “mhealth*”[tw] OR “mobile health app*”[tw] OR “mhealth intervention*”[tw] OR “mhealth app*”[tw] OR app OR “mobile intervention*”[tw] OR “smartphone intervention*”[tw] OR “smartphone app*”[tw] OR “mobile phone app*”[tw] OR “mobile phone intervention*”[tw] |  |
|  | 4 | "mobile applications"[mh] OR "Telemedicine"[mh] |  |
|  | 5 | “Quality of life”[tw] OR psycholog*[tw] OR mental*[tw] OR Anxiet*[tw] OR Anxious*[tw] OR Depress*[tw] OR Depressive*[tw] OR distress*[tw] OR “psychological stress*”[tw] OR self effica*[tw] |  |
|  | 6 | “Quality of life” [mh] OR “mental health” [mh] OR “Anxiety” [mh] OR “Anxiety disorders” [mh] OR “Depression” [mh] OR “depressive Disorder” [mh] OR “Mental disorders” [mh] OR “Stress, psychological” [mh] OR “self efficacy” [mh] |  |
|  | 7 | (#1 OR #2) AND (#3 OR #4) AND (#5 OR #6) |  |
|  | 8 | (#1 OR #2) AND (#3 OR #4) AND (#5 OR #6) Filters: Clinical Trial, Randomized Controlled Trial, English, Adult: 19+ years, Humans | **201** |
| 1. Scopus | 1 | ( TITLE-ABS-KEY ( neoplas* OR tumor* OR tumour* OR cancer* OR malignan* OR oncolog* OR leukemi* OR leukaemi* OR lymphoma* OR sarcoma* OR carcino* ) |  |
|  | 2 | TITLE-ABS-KEY ( "mobile app*" OR "software app*" OR "smartphone app*" OR "portable electronic app*" OR "mhealth*" OR "mobile health app*" OR "mhealth intervention*" OR "mhealth app*" OR app OR "mobile intervention*" OR "smartphone intervention*" OR "smartphone app*" OR "mobile phone app*" OR "mobile phone intervention*" ) |  |
|  | 3 | TITLE-ABS-KEY ( "Quality of life" OR psycholog* OR mental* OR anxiet* OR anxious* OR depress* OR depressive* OR distress* OR "psychological stress*" OR "self effica*" ) |  |
|  | 4 | #1 AND #2 AND #3 AND #4 |  |
|  | 5 | #1 AND #2 AND #3 AND #4 AND ( LIMIT-TO ( EXACTKEYWORD , "Human" ) ) AND ( LIMIT-TO ( EXACTKEYWORD , "Adult" ) ) AND ( LIMIT-TO ( EXACTKEYWORD , "Randomized Controlled Trial" ) ) | **122** |
| 4) Cochrane Library | 1 | (“Neoplas*” OR “Tumor*” OR “Tumour*” OR “Cancer*” OR “Malignan*” OR “Oncolog*” OR “Leukemi*” OR “Leukaemi*” OR “Lymphoma*” OR “Sarcoma*” OR “Carcino*”):ti,ab,kw |  |
|  | 2 | MeSH descriptor: [Neoplasms] explode all trees |  |
|  | 3 | MeSH descriptor: [Medical Oncology] explode all trees |  |
|  | 4 | (“mobile app*” OR “software app*” OR “smartphone app*” OR “portable electronic app*” OR “mhealth*” OR “mobile health app*” OR “mhealth intervention*” OR “mhealth app*” OR app OR “mobile intervention*” OR “smartphone intervention*” OR “smartphone app*” OR “mobile phone app*” OR “mobile phone intervention*”):ti,ab,kw |  |
|  | 5 | (MeSH descriptor: [Mobile Applications] explode all trees |  |
|  | 6 | MeSH descriptor: [Telemedicine] explode all trees |  |
|  | 7 | (“Quality of life” OR “Psycholog*” OR “mental health” OR “Anxiet*” OR “Anxious*” OR “Depress*” OR “Depressive*” OR “Distress*” OR “psychological stresses” OR “self effica*”):ti,ab,kw |  |
|  | 8 | MeSH descriptor: [Quality of Life] explode all trees |  |
|  | 9 | MeSH descriptor: [Mental Health] explode all trees |  |
|  | 10 | MeSH descriptor: [Anxiety] explode all trees |  |
|  | 11 | MeSH descriptor: [Anxiety Disorders] explode all trees |  |
|  | 12 | MeSH descriptor: [Depression] explode all trees |  |
|  | 13 | MeSH descriptor: [Depressive Disorder] explode all trees |  |
|  | 14 | MeSH descriptor: [Psychological Distress] explode all trees |  |
|  | 15 | MeSH descriptor: [Mental Disorders] explode all trees |  |
|  | 16 | MeSH descriptor: [Stress, Psychological] explode all trees |  |
|  | 17 | MeSH descriptor: [Self Efficacy] explode all trees |  |
|  | 18 | (#1 OR #2 OR #3) AND (#4 OR #5 OR #6) AND (#7 OR #8 OR #9 OR #10 OR #11 OR #12 OR #13 OR #14 OR #15 OR #16 OR #17) AND Filters: trails | **507** |
| 5) Web of science | 1 | (TS=(“Neoplas*” OR “Tumor*” OR “Tumour*” OR “Cancer*” OR “Malignan*” OR “Oncolog*” OR “Leukemi*” OR “Leukaemi*” OR “Lymphoma*” OR “Sarcoma*” OR “Carcino*”)) OR TS=(“Neoplasms” OR “Medical oncology”) |  |
|  | 2 | (TS=((“mobile app*” OR “software app*” OR “smartphone app*” OR “portable electronic app*” OR “mhealth*” OR “mobile health app*” OR “mhealth intervention*” OR “mhealth app*” OR app OR “mobile intervention*” OR “smartphone intervention*” OR “smartphone app*” OR “mobile phone app*” OR “mobile phone intervention*”):)) OR TS=(mobile application” OR “smartphone application” OR “telemedicine” OR “mhealth”) |  |
|  | 3 | (ALL=(“Quality of life” OR “Psycholog*” OR “mental health” OR “Anxiet*” OR “Anxious*” OR “Depress*” OR “Depressive*” OR “Distress*” OR “psychological stresses” OR “self effica*”)) OR ALL=(“Quality of life” OR “mental health” OR “psychological” OR “Anxiety” OR “Anxiety disorders” OR “Depression” OR “depression disorders” OR “distress” OR “psychological stresses” OR “self efficacy”) |  |
|  | 4 | TS= clinical trial* OR TS= randomised controlled trial* OR TS= controlled trial* OR TS=random* |  |
|  | 5 | #1 AND #2 AND #3 AND #4 | **287** |
| 6) CNKI | 1 | (主题=癌) OR (主题=肿瘤) |  |
|  | 2 | (主题=移动应用) OR (主题=应用程序) OR (关键词=远程应用) OR (关键词=手机应用) |  |
|  | 3 | (关键词=随机对照） |  |
|  | 4 | #1 AND #2 AND #3 | **4** |
| 7) Wan Fang | 1 | 主题:("癌症") or 主题:("肿瘤") |  |
|  | 2 | 主题:(移动医疗) or 主题:(移动健康) or 主题:(手机应用) |  |
|  | 3 | 主题:(随机对照实验) |  |
|  | 4 | #1 AND #2 AND #3 | **5** |
| TOTAL |  |  | **1491** |
