# Supplementary material for: Effect of Mobile Phone App–Based Interventions on Quality of Life and Psychological Symptoms Among Adult Cancer Survivors: Systematic Review and Meta-analysis of Randomized Controlled Trials
Source: J Med Internet Res. 2022 Dec 19;24(12):e39799. doi: 10.2196/39799 (PMC9808609; doi:10.2196/39799)
Supplement: Multimedia Appendix 2 [file jmir_v24i12e39799_app2.docx]

**The Supplement 2 for risk of bias**

| **No** | **Author, Publication year** | **Random sequence generation (selection bias)** | **Allocation concealment (selection bias)** | **Blinding of participants and personnel (performance bias)** | **Blinding of outcome assessment (detection bias)** | **Incomplete outcome data (attrition bias)** | **Selective reporting (reporting bias)** | **Other bias** | **Overall risk of bias** |
| --- | --- | --- | --- | --- | --- | --- | --- | --- | --- |
| 1 | Absolom et al [44], 2021 | Low risk | Unclear | high risk | high risk | Unclear | Low risk | Low risk | Unclear |
| 2 | Kubo et al [16], 2020 | Low risk | Unclear | Unclear | high risk | Low risk | Low risk | Low risk | Low risk |
| 3 | Karaaslan-Eser and Ayaz-Alkaya [31], 2021 | Unclear | Low risk | High risk | high risk | Low risk | Low risk | High risk | Low risk |
| 4 | Berg et al [45], 2019 | Low risk | Unclear | Unclear | high risk | Low risk | Low risk | Unclear | Unclear |
| 5 | Huggins et al [47], 2022 | Low risk | Unclear | High risk | high risk | Unclear | Unclear | Unclear | high risk |
| 6 | Seib et al [49], 2022 | Low risk | High risk | High risk | high risk | Low risk | Low risk | Low risk | High risk |
| 7 | Chen et al [46], 2021 | Low risk | Unclear | Unclear | high risk | Low risk | Low risk | Unclear | Low risk |
| 8 | Çınar et al [26], 2021 | Unclear | Low risk | Unclear | Unclear | Unclear | Low risk | Unclear | Unclear |
| 9 | Ghanbari et al [17], 2021 | Low risk | Unclear | High risk | Unclear | High risk | Low risk | Unclear | Unclear |
| 10 | Børøsund et al [25], 2021 | Low risk | Unclear | High risk | Unclear | Low risk | Low risk | High risk | Unclear |
| 11 | Zha [43], 2020 | Low risk | High risk | High risk | high risk | Low risk | Unclear | Unclear | High risk |
| 12 | Kim et al [19], 2018 | Low risk | Unclear | High risk | High risk | Low risk | Low risk | Unclear | Low risk |
| 13 | Park et al [32], 2021 | Low risk | Low risk | Unclear | Unclear | Unclear | High risk | Unclear | Unclear |
| 14 | Hou et al [40], 2020 | Low risk | Low risk | Unclear | Unclear | Low risk | Low risk | Low risk | Unclear |
| 15 | Zhu et al [37], 2018 | Low risk | Low risk | High risk | high risk | Low risk | Unclear | Low risk | Unclear |
| 16 | Greer et al [29], 2020 | Low risk | Low risk | High risk | Unclear | Low risk | Low risk | Low risk | Unclear |
| 17 | Greer et al [28], 2019 | Low risk | Unclear | High risk | Unclear | Unclear | Low risk | Low risk | Unclear |
| 18 | Zhou et al [36], 2019 | Low risk | Unclear | High risk | high risk | Unclear | Low risk | Low risk | Unclear |
| 19 | Rosen et al [42], 2018 | Low risk | Low risk | High risk | high risk | Unclear | High risk | Unclear | High risk |
| 20 | Ham et al [30], 2019 | Low risk | Unclear | High risk | Unclear | Low risk | Unclear | Low risk | Low risk |
| 21 | Lei [41], 2016 | Low risk | Unclear | High risk | Unclear | Low risk | Unclear | Unclear | Low risk |
| 22 | Maguire et al [48], 2021 | Low risk | Low risk | High risk | Unclear | Unclear | Low risk | Unclear | Unclear |
| 23 | Fjell et al [27], 2020 | Low risk | Low risk | Unclear | high risk | Low risk | Low risk | Low risk | Unclear |
| 24 | Foley et al [20], 2016 | Unclear | Low risk | Unclear | high risk | Low risk | Unclear | High risk | High risk |
| 25 | Di and Li [38], 2018 | Low risk | High risk | High risk | Unclear | Low risk | Unclear | Unclear | High risk |
| 26 | Handa et al [18], 2020 | Low risk | Unclear | Unclear | Unclear | Low risk | Low risk | Low risk | Unclear |
| 27 | Spahrkäs et al [34], 2020 | Low risk | Low risk | Unclear | Unclear | Unclear | Low risk | Low risk | Unclear |
| 28 | Dong et al [39], 2019 | Low risk | Low risk | High risk | Unclear | Unclear | Unclear | Low risk | Low risk |
| 29 | Sui et al [35], 2020 | Low risk | Low risk | High risk | high risk | High risk | Unclear | Unclear | Unclear |
| 30 | Peng et al [33], 2020 | Low risk | Unclear | Unclear | Unclear | Low risk | Low risk | Low risk | Low risk |
