# Supplementary figures and images for: Effect of Mobile Phone App–Based Interventions on Quality of Life and Psychological Symptoms Among Adult Cancer Survivors: Systematic Review and Meta-analysis of Randomized Controlled Trials
Source: J Med Internet Res. 2022 Dec 19;24(12):e39799. doi: 10.2196/39799 (PMC9808609; doi:10.2196/39799)

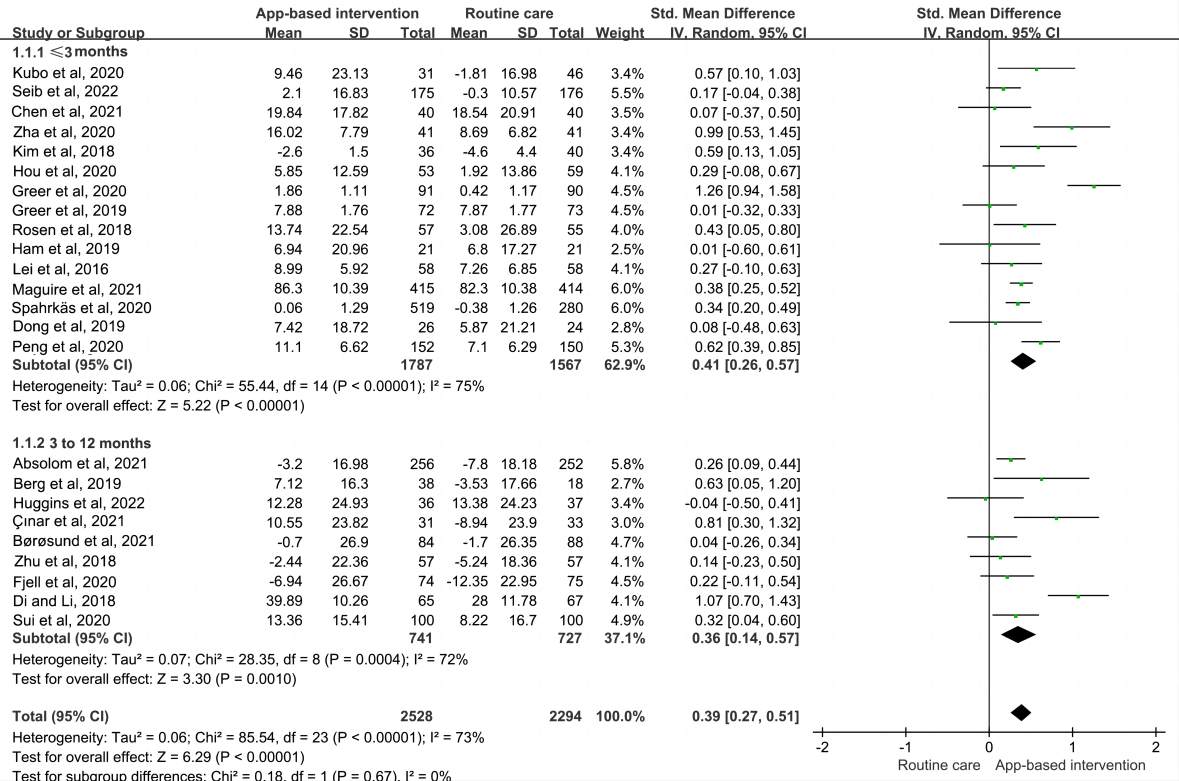

Supplement: Multimedia Appendix 3 [file jmir_v24i12e39799_app3.png]

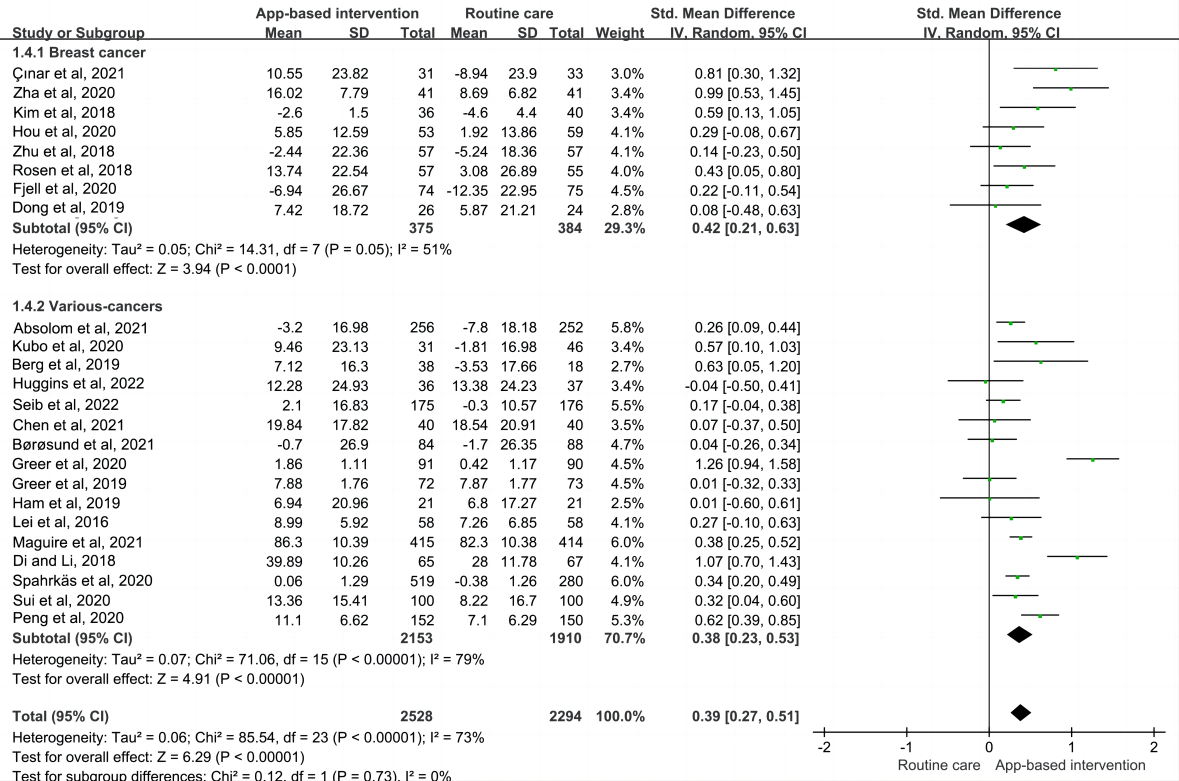

Supplement: Multimedia Appendix 4 [file jmir_v24i12e39799_app4.png]

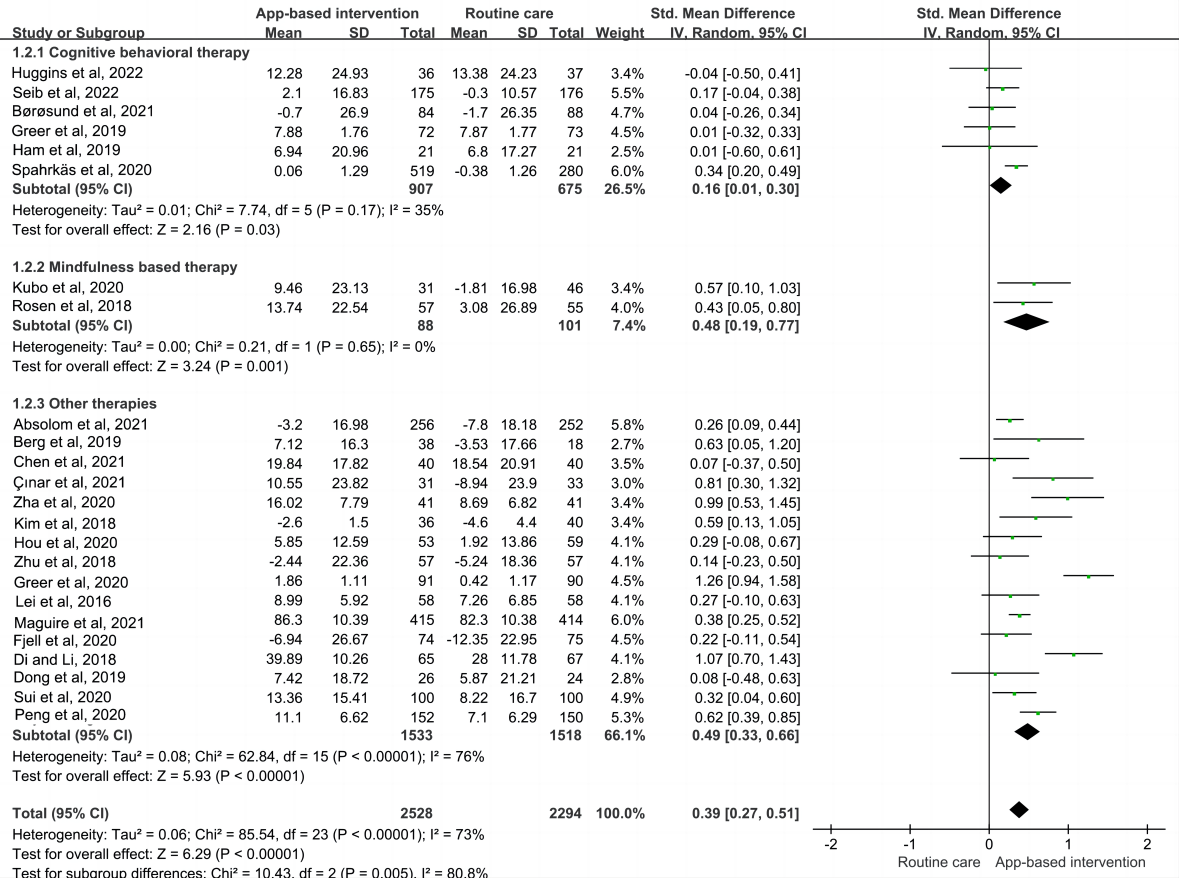

Supplement: Multimedia Appendix 5 [file jmir_v24i12e39799_app5.png]

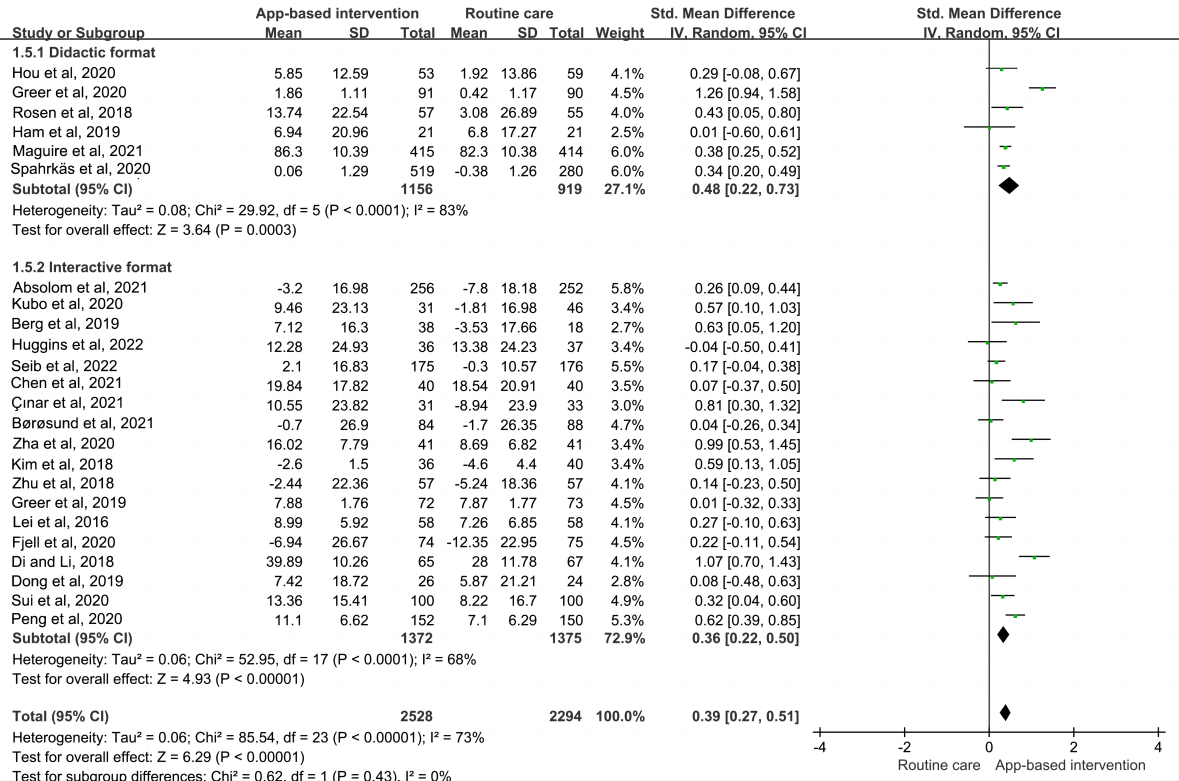

Supplement: Multimedia Appendix 6 [file jmir_v24i12e39799_app6.png]

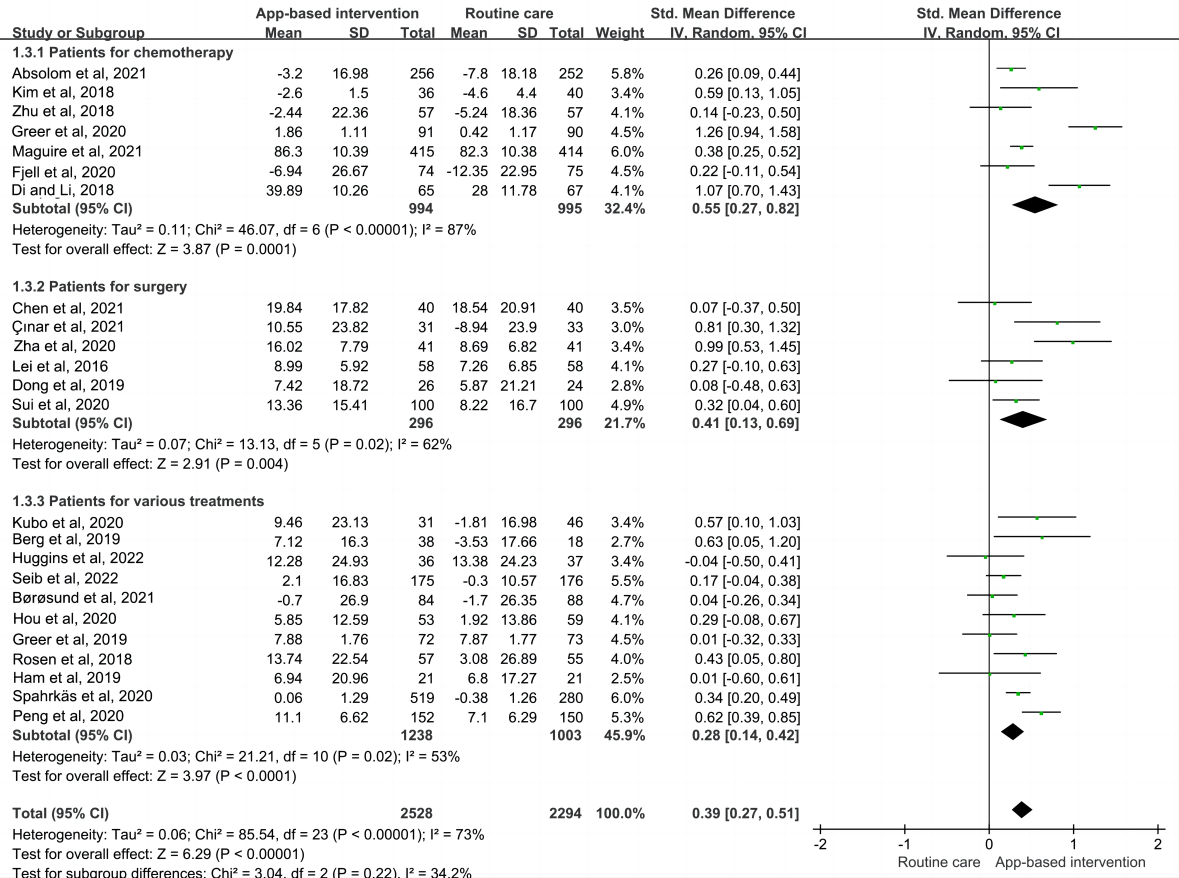

Supplement: Multimedia Appendix 7 [file jmir_v24i12e39799_app7.png]

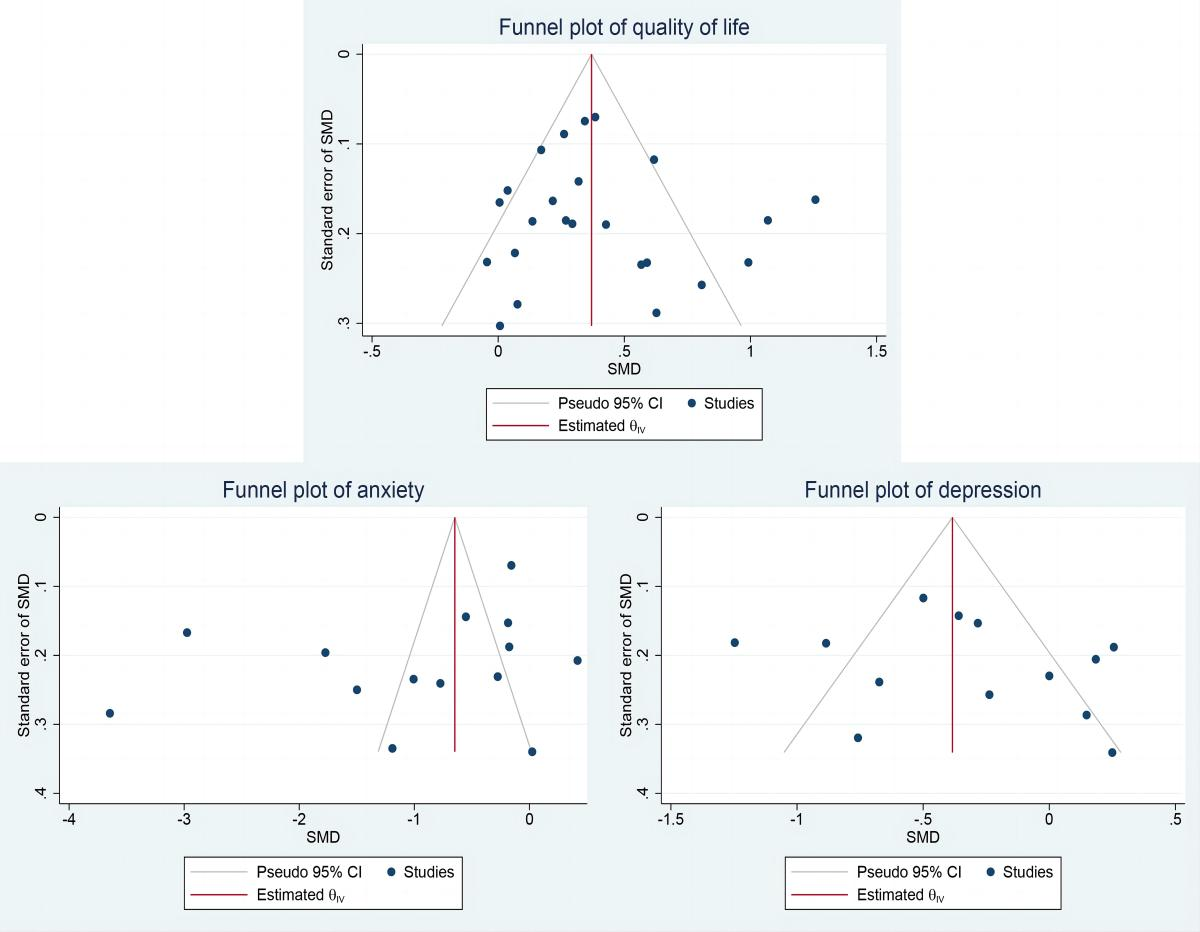

Supplement: Multimedia Appendix 8 [file jmir_v24i12e39799_app8.png]
